# Amplitudes of cortical somatosensory evoked potentials and outcome prediction after cardiac arrest

**DOI:** 10.1186/cc14516

**Published:** 2015-03-16

**Authors:** C Storm, CJ Ploner, C Leithner

**Affiliations:** 1 Charite Universitaetsmedizin, Berlin, Germany

## Introduction

Bilaterally absent cortical somatosensory evoked potentials (SSEPs) predict poor outcome after cardiac arrest. A threshold for the amplitude of early cortical SSEPs above which patients may survive with good outcome has not been determined. Thus, tolerable noise levels for the interpretation of cortical SSEPs are poorly defined. Furthermore, it has not been systematically investigated whether high amplitudes of cortical SSEPs may exclude severe hypoxic encephalopathy incompatible with re-awakening.

## Methods

We prospectively studied SSEPs after median nerve stimulation obtained 24 hours to 4 days after cardiac arrest in patients treated with targeted temperature management at 33°C for 24 hours. Amplitudes of cortical SSEPs were determined, if at least two peripheral, spinal and cortical recordings per side were available, spinal potentials were bilaterally reproducible and cortical noise level was below 0.25 μV. Cortical SSEP amplitude was defined as largest amplitude of a reproducible cortical SSEP of four cortical recordings (two per side) within 50 milliseconds after stimulation. Outcome was assessed upon ICU discharge using the Cerebral Performance Category (CPC) scale. CPC 1 to 3 was defined as good outcome, CPC 4 to 5 as poor outcome.

## Results

Of 318 consecutive patients examined, 293 had complete SSEP recordings with reproducible spinal potentials and cortical noise levels below 0.25 μV. Of those, 137 (47%) had good outcome and 156 (53%) had poor outcome. The lowest amplitude of the early cortical SSEPs in a survivor with good outcome was 0.62 μV. All 79 patients with amplitudes below this threshold had poor outcome. None of 27 patients who survived with CPC 4 (unresponsive wakefulness syndrome) had cortical SSEP amplitudes above 2.5 μV. Twenty-four patients with amplitudes above this limit died. Detailed case review indicated a cause of death other than hypoxic encephalopathy in these patients.

## Conclusion

Our data indicate that the prognostic value of SSEP after cardiac arrest extends beyond a mere absent-present dichotomy. Bilaterally absent as well as very low amplitude SSEPs predict poor outcome with high positive predictive value. SSEPs should not be used for prognostication, if noise in cortical recordings could mask critically low amplitudes. High amplitudes of early cortical SSEPs strongly argue against severe hypoxic encephalopathy incompatible with re-awakening.

